# Assessment of Drought Tolerance Degree (DTD) method as a reliable tool for early-stage screening for drought tolerance in *indica* rice

**DOI:** 10.1186/s12870-025-07591-7

**Published:** 2025-11-24

**Authors:** Sandeep Kumar Singh, Jawahar Lal Katara, C. Parameswaran, Prem Narayan Jagadev, Debendra Nath Bastia, Kishor Jeughale, Sanghamitra Samantaray

**Affiliations:** 1 Department of Genetics and Plant Breeding, Faculty of Agricultural Sciences, Siksha ‘O’ Anusandhan University, Bhubaneswar, Odisha 751030 India; 2 Crop Improvement Division, ICAR-National Rice Research Institute, Cuttack, Odisha 753006 India; 3 Department of Plant Breeding and Genetics, Odisha University of Agriculture and Technology, Bhubaneswar, Odisha 751003 India

**Keywords:** Drought tolerance, Drought tolerance degree (DTD), Doubled haploid lines, Abiotic stress, Indica rice, Rice drought screening, RWC, Leaf rolling, Leaf tip drying

## Abstract

Drought stress poses a significant threat to rice production, particularly in indica cultivars that form the staple diet for a large portion of the world's population. Efficient and reliable screening methods are essential to accelerate the development of drought-tolerant rice varieties. The objective of the study was to assess and validate the efficacy of the Drought Tolerance Degree (DTD) method for early-stage drought tolerance screening in a diverse population of 118 doubled haploid (DH) indica rice lines and their parents. Plants were subjected to controlled severe drought stress under pot culture in a net house environment, and drought responses were assessed using DTD method alongside key physiological traits including relative water content (RWC), chlorophyll content index, leaf rolling and drying scores, leaf canopy temperature, leaf area, tiller and leaf numbers, and plant height. The DTD values exhibited strong positive correlations with RWC (*r* = 0.771) and chlorophyll content (*r* = 0.526), and strong negative correlations with leaf rolling (*r* = -0.850), leaf drying scores (*r* = -0.778), canopy temperature, and tiller number. Principal component and hierarchical clustering analyses further confirmed the association of DTD with drought tolerance-related traits and effectively discriminated tolerant and susceptible genotypes. In comparison with traditional methods, the DTD assay is cost-effective, rapid, and requires minimal technical expertise, making it practical for high-throughput screening in breeding programs. However, its applicability is limited to early growth stages due to the confounding effects of natural leaf senescence at maturity. Overall, this work demonstrates the reliability and efficiency of the DTD method in assessing drought tolerance in indica rice, offering a valuable phenotyping tool to facilitate the selection of drought-resilient cultivars in breeding pipelines.

**Supplementary Information:**

The online version contains supplementary material available at 10.1186/s12870-025-07591-7.

## Introduction

Rice is a staple food for billions of people around the globe, playing a vital role in Asia, where it accounts for much of the daily calorie and protein intake [1, 2]. In many developing countries, rice helps supply essential dietary energy and protein, making it a cornerstone of food security [2]. Traditionally, rice grows best in regions with abundant water and reliable rains, but to secure the demands of the world’s population projected to rise to 8.6 billion by 2030, boosting rice production is more crucial than ever [3].

One of the toughest challenges faced by rice farmers is drought [4, 5]. When water becomes scarce, rice plants, especially the high-yielding varieties that have been developed for modern farming, are quick to feel the effects [6, 7]. Shortages in rainfall or limited irrigation can reduce crop yields dramatically, threatening livelihoods and local economies. Globally, more than 20 million hectares of rice fields are vulnerable to drought each year, especially in rainfed regions where farmers rely solely upon the weather [8, 9]

To help secure harvests against drought, researchers focus on breeding rice varieties that can endure water shortages. This effort depends on two major factors: first, finding and using rice strains that naturally cope with drought; and second, having reliable ways to measure how well different rice varieties withstand dry conditions, ideally, methods that are practical for breeders and effective at large scales [10, 11].

Although many approaches have been tested to evaluate drought resilience, some are dependent upon technical measurements or complex lab skills, which can be time-consuming and expensive. Farmers and breeders urgently need faster and easier ways to identify drought-tolerant rice at early growth stages, so that the best lines can be advanced to breeding programs.

This study aims to address that need by adapting and validating a relatively simple screening tool, known as the Drought Tolerance Degree (DTD) method, originally proposed by Zu et al. [12] for use in rice at the early stages of growth. By focusing on a method that is both efficient and accessible, this work hopes to make it easier to identify rice varieties that can survive and thrive in water-deficient conditions.

## Methods

### Experimental site and plant material

This study was conducted under controlled conditions in a net house at the ICAR–National Rice Research Institute, Cuttack, Odisha, India (20°27′9″N, 85°56′25″E). The experimental material consisted of 118 doubled haploid lines, developed via androgenesis from the F₁ progeny of a cross between two parent varieties: IR-20 (drought susceptible) and Mahulata (drought tolerant) (Chakraborty, K. et al. [13]. Trait Specific Donors for Rice Improvement: A Compendium. ICAR-NRRI, Cuttack 2018; [14]). The two parental lines, maintained as part of the germplasm collection at ICAR–National Rice Research Institute, Cuttack, India, were also included and served as experimental controls. The DH mapping population was developed to identify the QTLs/genes for drought tolerance at the vegetative stage at this institute, and details of its development and characterization are described in our previous publication [15].

Seedlings were transplanted into earthen pots (30 cm height × 30 cm diameter) filled with homogenized lowland soil collected from the NRRI farm. In each pot, dry soil was added in 5 cm increments and compacted using a wooden rod. Bulk density of the packed soil was verified at each step using a metal core sampler (100 cm^3^ volume), and the dry weight was measured after oven drying at 105 °C for 24 h. This procedure was repeated until a uniform bulk density of approximately 1.15 g/cm^3^ was achieved throughout the lower 80% of the soil profile, following Bernier et al. [16]. Before transplanting, the soil in the pots was saturated with water for several days to facilitate settling. The upper 20% of each pot was topped with irrigated lowland soil, leaving the soil surface 5 cm below the rim. Drainage holes at the bottom of the pots were sealed to prevent water loss. All genotypes, including both parents, were grown in three replicates to minimize environmental effects. Throughout the study, the net house was kept fully protected from rainfall to maintain consistent soil moisture conditions.

### Drought stress treatment

After transplanting, all pots received irrigation every other day until the start of the drought treatment. Initial soil moisture at a depth of 20 cm was maintained at approximately 56% (v/v) as measured by a moisture meter. Drought stress was imposed 11 days after transplanting by withholding further irrigation. Throughout the drought stress period, the susceptible control plants received regular irrigation, along with the tolerant control, to maintain optimal soil moisture levels. This ensured reliable comparison between drought-treated plants and both controls, clarifying drought-specific responses in the experimental lines. Soil moisture content was monitored at a 20 cm depth using a digital soil moisture meter (Model: IRROMETER WATERMARK 200SS, Spectrum Technologies Inc., USA). The meter was calibrated before the experiment by correlating its digital readings with gravimetric soil moisture values measured on representative samples dried at 105 °C for 24 h. Meter readings consistently matched gravimetric estimates within ± 2% throughout the experiment. Soil moisture content continued to be monitored on alternate days using the same moisture meter. Soil water potential at a depth of 15 cm was measured on alternate days using tensiometers (Soil Moisture Equipment Co.), which were randomly inserted at five to six locations in both stressed and control pots. Drought treatment was continued until visual symptoms of drought stress, such as leaf damage in the tolerant parent (Mahulata), became prominent. This approach enabled the assessment of the drought response, particularly in lines that potentially exceeded the parental tolerance.

### Data collection

Soil moisture content and water potential readings were recorded every other day throughout the stress period. Leaf tip drying and leaf rolling were assessed according to the Standard Evaluation System (SES) [17]. Canopy temperature was measured using an infrared thermometer (Model IR50, Spectrum Instrument Ltd.), and chlorophyll content was determined using a SPAD-502 m. The relative water content (RWC) of the leaves was measured on the last day of the drought treatment at 2 pm, following the method of Matin et al. [18]. Leaf area (cm^2^) was calculated as: leaf length × leaf width × 0.71 [19]. Additional data, including plant height, number of leaves, and number of tillers per plant, were also recorded at the end of the drought period. All physiological measurements, such as relative water content, chlorophyll content, canopy temperature, leaf rolling, and leaf tip drying, were conducted on the last day of drought stress (day 15) only on plants and genotypes that were still alive. This method ensured accurate assessment of traits by excluding plants that were completely dried out or no longer viable. Plants that had died prior to the measurement were excluded from the physiological evaluations. All observations were made on the final day of the stress treatment.

### Calculation of Drought Tolerance Degree (DTD)

**Fig. 1:**
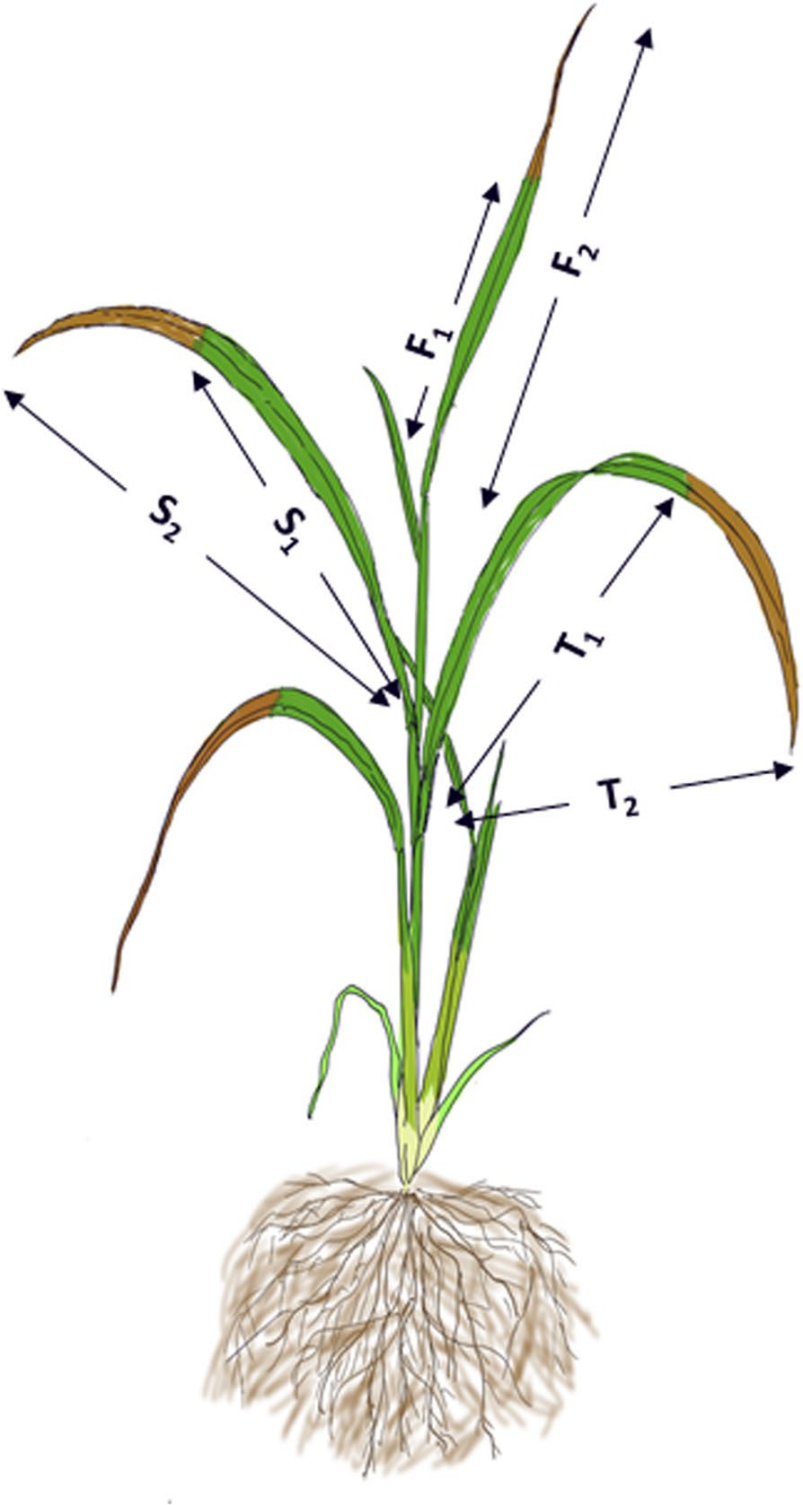
Visualization of the Drought Tolerance Degree (DTD) measurement method showing green and total leaf portions after severe drought stress. F1, S1, and T1 indicate the lengths of the green (healthy) parts of the first, second, and third leaves, respectively, while F2, S2, and T2 indicate the corresponding total leaf lengths. The brown-colored sections represent dried parts of the leaves caused by drought-induced damage. The DTD is calculated as the average ratio of green leaf length to total leaf length, providing a quantitative measure of drought tolerance based on leaf retention

The Drought Tolerance Degree (DTD) method, as described by Zu et al. [12], was used to evaluate drought response. DTD is defined as the average proportion of green leaf length to total leaf length for the top three leaves of each plant after exposure to severe drought stress (Fig. 1). Values for DTD range from 0 to 1, where higher values indicate greater drought tolerance.

Green and total leaf lengths were measured for each of the top three leaves (denoted as F1/F2 for the first leaf, S1/S2 for the second, and T1/T2 for the third). The control (well-watered) plants were evaluated in the same manner for comparative purposes.

The DTD for each plant was calculated using the following formula:

$$Xj=\frac1nn\Sigma\left(i=1\right)\left[\left(\frac{F1}{F2}+\frac{S1}{S2}+\frac{T1}{T2}\right)/3\right]$$

DTD value = (X_1_ + X_2_ + X_3_)/3.

where, n is the number of measured plants in each replicate, Xj represents one of the three replicates DTD value in each cultivar, X_1_, X_2_, X_3_ denote replicate I, replicate II, and replicate III, respectively.

### Statistical analysis

All the data collected were subjected to analysis of PCA, correlation study, and hierarchical analysis by using PAST 4.03.

## Results

### Responses of 118 doubled haploid rice lines to drought stress

**Fig. 2:**
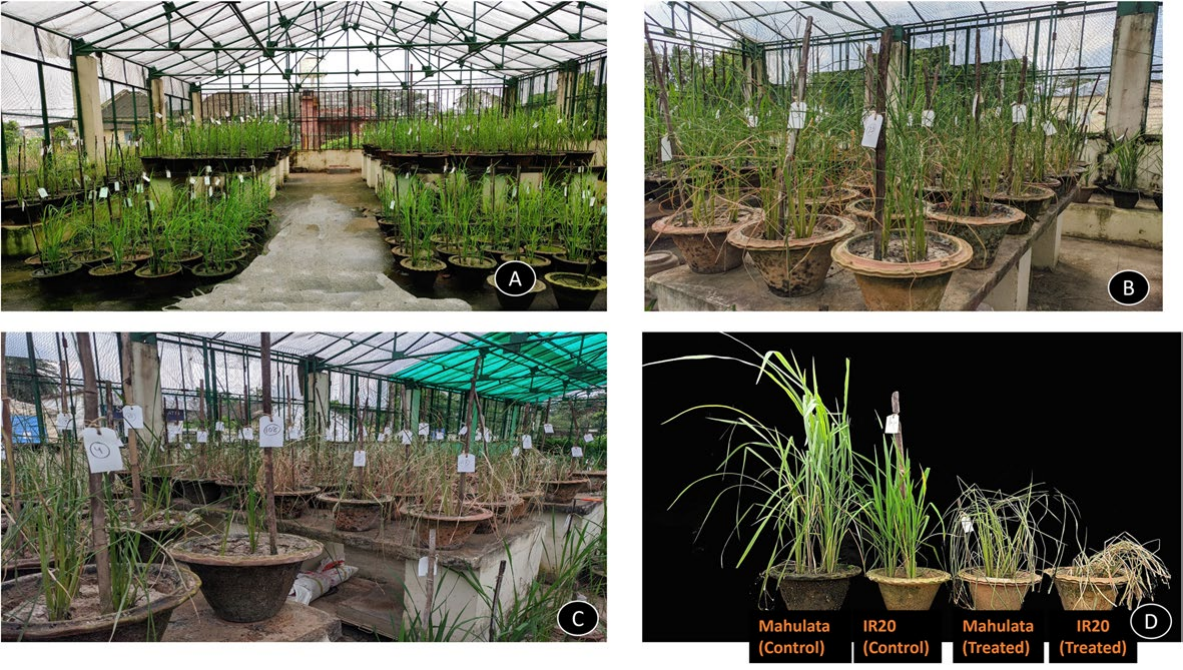
Response of 118 doubled haploid indica rice lines and their parents to various water treatments. **A** Visual performance of all experimental lines grown under well-watered conditions. **B** and **C** Phenotypic responses of the experimental population on the final day (Day 15) of drought treatment, showing variability in drought tolerance among lines. **D** Comparative drought response of the two parental varieties, Mahulata (drought-tolerant) and IR-20 (drought-sensitive), under both well-watered (control) and drought stress conditions on the last day of treatment (Day 15 of drought treatment)

**Fig. 3:**
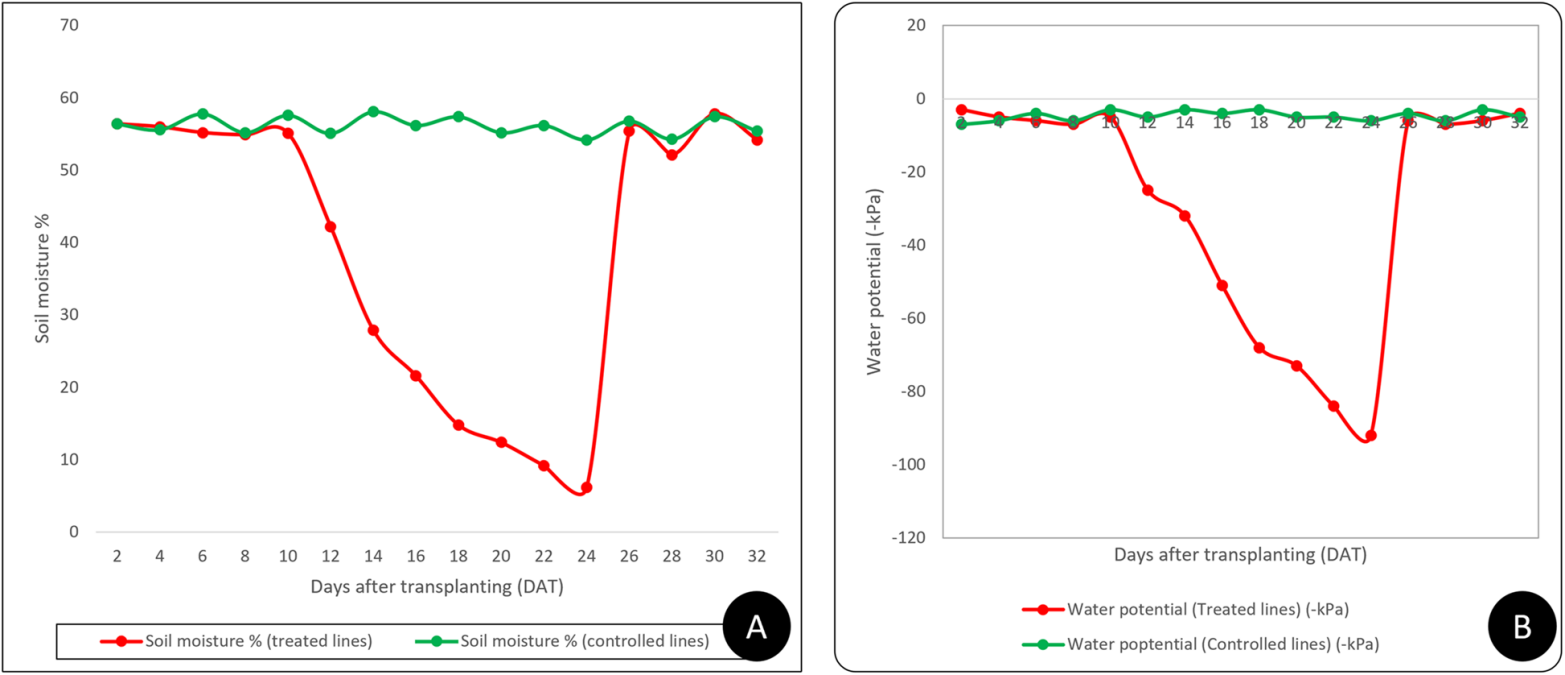
**A** Soil moisture percentage and B) soil water potential (kPa) dynamics recorded in drought-stressed and well-watered control indica rice populations throughout the experimental period. Soil moisture at 20 cm depth was measured every other day using a moisture meter, while soil water potential at 15 cm depth was recorded on alternate days using tensiometer tubes (Soil Moisture Equipment Co.). Drought stress was imposed by stopping irrigation 12 days after transplanting (DAT). Soil readings correspond to 7, 12, and 15 days after irrigation was withheld, coinciding with the 17th, 21 st, and 24th DAT, respectively. On the 24th day after transplanting (the final day of drought treatment), the pots were rewatered (after collecting all the phenotypic data) to restore soil moisture levels

Before the onset of drought stress, all 118 doubled haploid (DH) rice lines, along with their two parental lines, grew well in earthen pots within the protected net house (Fig. 2). None of them exhibited signs of leaf rolling or tip drying, indicating healthy initial growth. However, as soil moisture at 15 cm depth dropped to approximately 15% and soil water potential dropped to −73 kPa, seven days after irrigation was stopped (Approx 18–19 days after transplanting), some differences in drought response became evident (Fig. 3). Specifically, DH-22 and DH-5 exhibited severe leaf rolling and drying of leaf tips, indicating that they are the most drought-sensitive lines in the panel (Table 1; these two lines have the lowest DTD). The remaining DH lines and both parents remained largely unaffected at this stage. As drought stress intensified, with soil moisture falling to 10% and soil water potential falling to −84 kPa twelve days after water cessation (moderate drought stress) (Fig. 3), additional lines such as DH-134, DH-120, DH-83, DH-75, DH-60, and DH-52 exhibited symptoms like tight leaf rolling, wilting, and drying, indicating susceptibility to drought. Under severe drought stress, which arose 14 days after watering was withheld (24–25 days after transplanting), when soil moisture reached around 5–6% and soil water potential dropped to −92 kPa (Fig. 3), significant damage was observed across most genotypes. Lines DH-5 and DH-22 were completely dead, whereas DH-44, DH-95, DH-102, and DH-126 demonstrated markedly less leaf damage, suggesting improved drought tolerance. Supporting this, these tolerant lines consistently showed lower scores for leaf rolling and leaf tip drying, along with higher relative water content (RWC), reinforcing their status as drought-tolerant cultivars. The control plants, which included both the drought-tolerant and susceptible parental lines maintained under regular irrigation, showed no signs of leaf rolling, tip drying, or other drought-related symptoms throughout the stress period (Table 1; control lines having the highest DTD values). Their foliage remained healthy and unaffected, distinguishing them clearly from the drought-stressed lines.

**Table 1:** Drought Tolerance Degree (DTD) values of 118 doubled haploid indica rice lines and parental controls under severe drought stress

Genotype Name	Mean DTD	SD (±)	Genotype Name	Mean DTD	SD (±)	Genotype Name	Mean DTD	SD (±)
DH1	0.663	0.130	DH54	0.856	0.007	DH100	0.298	0.002
DH3	0.548	0.030	DH56	0.424	0.099	DH101	0.989	0.007
DH4	0.419	0.050	DH57	0.761	0.022	DH102	0.994	0.001
DH5	0.007	0.000	DH58	0.679	0.018	DH104	0.182	0.080
DH6	0.232	0.041	DH59	0.948	0.021	DH106	0.522	0.102
DH7	0.458	0.133	DH60	0.016	0.003	DH107	0.770	0.005
DH8	0.639	0.121	DH62	0.057	0.004	DH108	0.607	0.083
DH9	0.198	0.035	DH64	0.982	0.006	DH109	0.313	0.212
DH10	0.910	0.077	DH65	0.344	0.182	DH110	0.098	0.007
DH12	0.988	0.007	DH66	0.087	0.002	DH113	0.952	0.049
DH13	0.989	0.005	DH67	0.130	0.020	DH114	0.363	0.215
DH14	0.981	0.009	DH69	0.663	0.153	DH115	0.680	0.046
DH15	0.656	0.205	DH70	0.663	0.153	DH116	0.635	0.023
DH16	0.617	0.220	DH71	0.680	0.098	DH117	0.586	0.305
DH17	0.186	0.081	DH73	0.073	0.014	DH118	0.123	0.023
DH18	0.288	0.079	DH74	0.544	0.052	DH119	0.056	0.012
DH20	0.989	0.005	DH75	0.086	0.003	DH120	0.046	0.009
DH22	0.007	0.001	DH76	0.496	0.001	DH121	0.380	0.046
DH25	0.285	0.055	DH77	0.388	0.071	DH122	0.060	0.020
DH28	0.574	0.118	DH78	0.550	0.045	DH123	0.084	0.024
DH29	0.623	0.006	DH79	0.059	0.024	DH124	0.509	0.251
DH30	0.507	0.029	DH80	0.983	0.003	DH125	0.500	0.129
DH31	0.671	0.003	DH81	0.286	0.124	DH126	0.991	0.000
DH32	0.542	0.010	DH82	0.710	0.133	DH127	0.988	0.002
DH34	0.740	0.011	DH83	0.154	0.022	DH128	0.630	0.106
DH36	0.197	0.065	DH84	0.238	0.013	DH129	0.662	0.045
DH37	0.055	0.001	DH85	0.273	0.113	DH130	0.869	0.051
DH38	0.563	0.044	DH86	0.099	0.009	DH131	0.547	0.034
DH39	0.552	0.007	DH87	0.267	0.044	DH132	0.240	0.104
DH40	0.384	0.062	DH88	0.250	0.118	DH133	0.516	0.128
DH41	0.988	0.007	DH89	0.836	0.093	DH134	0.022	0.004
DH43	0.772	0.045	DH90	0.983	0.004	DH135	0.072	0.017
DH44	0.992	0.000	DH91	0.286	0.082	DH136	0.626	0.305
DH45	0.397	0.084	DH94	0.625	0.038	DH137	0.986	0.007
DH46	0.150	0.079	DH95	0.990	0.002	Mahulata	0.639	0.033
DH48	0.269	0.182	DH96	0.056	0.058	IR20	0.014	0.007
DH50	0.098	0.012	DH97	0.202	0.010	Mahulata(Control)	0.993	0.003
DH51	0.994	0.000	DH98	0.122	0.037	IR20(Control)	0.989	0.004
DH52	0.020	0.002	DH99	0.092	0.007			

### Drought Tolerance Degree (DTD) values after severe drought

The Drought Tolerance Degree (DTD) index quantifies drought tolerance based on the ratio of green leaf length to total leaf length of the top three leaves, with higher values indicating less drought-induced damage. Under well-watered conditions, all genotypes displayed similar, high DTD values (~ 0.99), consistent with healthy plants. Following a severe drought, DH-102 recorded the highest DTD value (0.993), closely followed by DH-44 (0.992), DH-126 (0.990), and DH-95 (0.990), highlighting their strong drought tolerance (Table 1). At the other extreme, DH-22 had the lowest DTD (0.006), even less than the drought-susceptible parent IR-20 (0.014), confirming its high vulnerability (Table 1). Notably, Mahulata, the drought-tolerant parental variety, showed a DTD of 0.639, considerably lower than DH-102, indicating that the latter outperformed even the known tolerant parent. These DTD results align closely with visual stress symptoms and physiological data collected during the drought trials, confirming that the DTD index is a reliable and objective measure of drought tolerance in this population.

### The correlation of DTD values with physiological traits

**Fig. 4:**
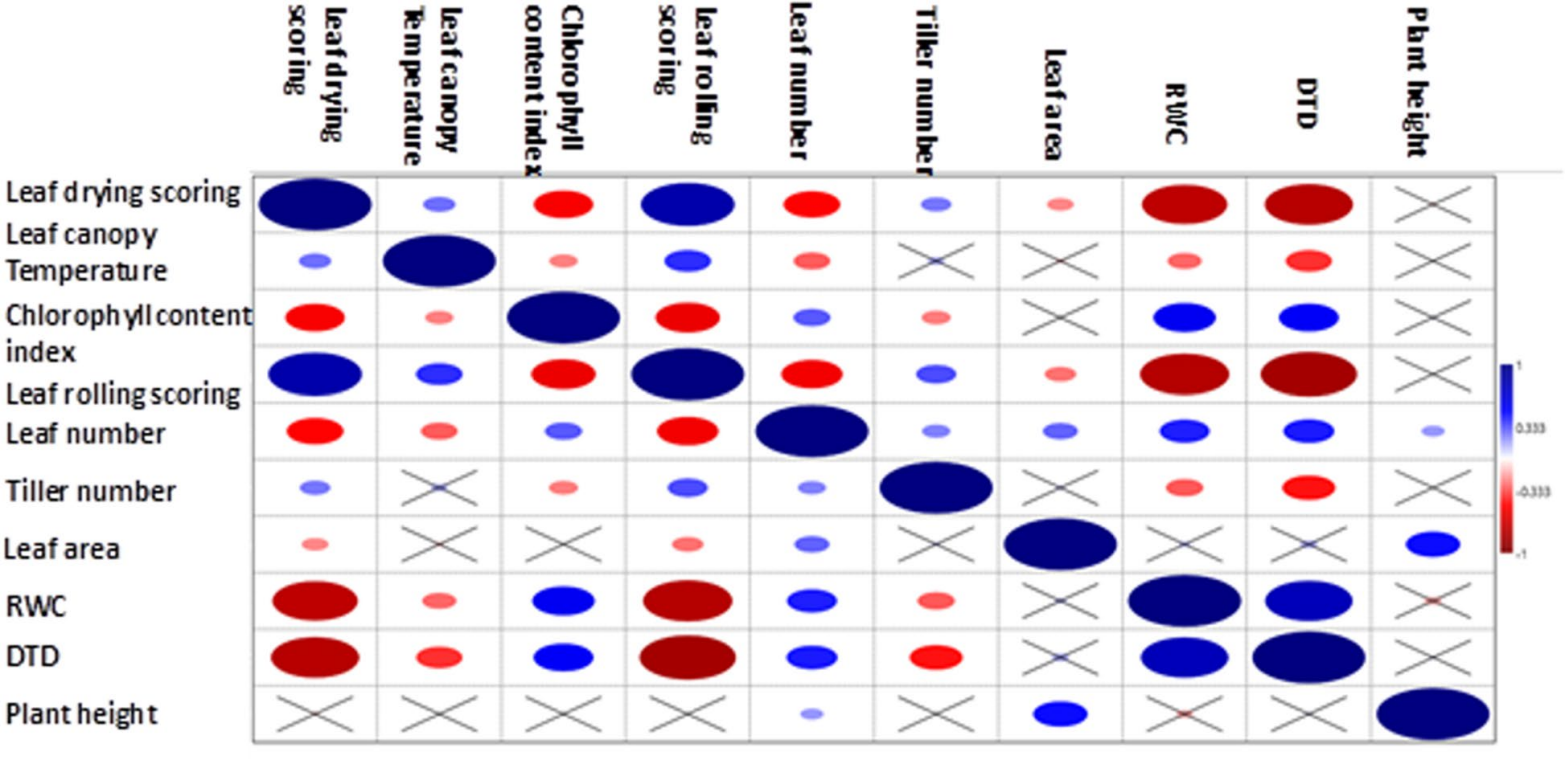
Schematic representation of correlation analysis among measured physiological and morphological traits. Diagram illustrating the relationships and strength of correlations between drought tolerance–related traits assessed in this study, including Drought Tolerance Degree (DTD), relative water content (RWC), leaf rolling, leaf drying, chlorophyll content index, leaf area, tiller number, leaf canopy temperature, and others

Previous studies have shown that several physiological traits closely correspond with drought tolerance in rice [20]. In this study, the Drought Tolerance Degree (DTD) exhibited a strong positive correlation with relative water content (RWC) (*r* = 0.771) (Table 2, Fig. 4). Conversely, DTD showed strong negative correlations with leaf drying score (*r* = −0.778) and leaf rolling score (*r* = −0.850), indicating that higher DTD values are associated with less visible drought-induced leaf damage.

**Table 2:** Pearson correlation coefficients between Drought Tolerance Degree (DTD) and morphophysiological traits in indica rice under drought stress

	LDS	LCT	CCI	LRS	LN	TN	LA	RWC	DTD	PH
LDS	1	0.2005*	−0.52242*	0.82642**	−0.49793*	0.26403*	−0.23101*	−0.75324**	−0.7785**	−0.06398
LCT		1	−0.24507*	0.40989*	−0.31919*	0.14586	−0.08317	−0.29677*	−0.4056*	−0.011496
CCI			1	−0.56678*	0.32372*	−0.252214*	0.039029	0.5489*	0.52619*	0.048541
LRS				1	−0.53969*	0.35035*	−0.27265*	−0.78802**	−0.85088**	−0.00872
LN					1	0.24395*	0.30444*	0.43929*	0.44776*	0.19921
TN						1	0.077606	−0.32479*	−0.46501*	0.000.87
LA							1	0.10226	0.14855	0.47744*
RWC								1	0.077171**	−0.15177
DTD									1	0.056831
PH										1

*LDS* Leaf drying scoring, *LCT* Leaf canopy temperature (^0^C), *CCI* Chlorophyll content index, *LRS* Leaf rolling scoring, *LN* leaf number, *TN* Tiller number, *LA* Leaf area (cm^2^), *RWC* Relative water content, *DTD* Drought tolerance degree, *PH* Plant height(cm)

^*^,**Significant at 0.05 and 0.01 level respectively

Additionally, DTD showed a positive correlation with the chlorophyll content index (*r* = 0.526) and leaf number (*r* = 0.447), while exhibiting negative correlations with both leaf canopy temperature (*r* = −0.405) and tiller number (*r* = −0.465). In consistent with these findings, RWC was strongly negatively correlated with leaf drying score (*r* = −0.753) and leaf rolling score (*r* = −0.788). Moreover, leaf drying and leaf rolling scores were highly positively correlated with one another (*r* = 0.826), further validating their use as complementary indicators of drought stress severity.

### Principal Component Analysis (PCA) of DTD and other physiological traits

**Fig. 5:**
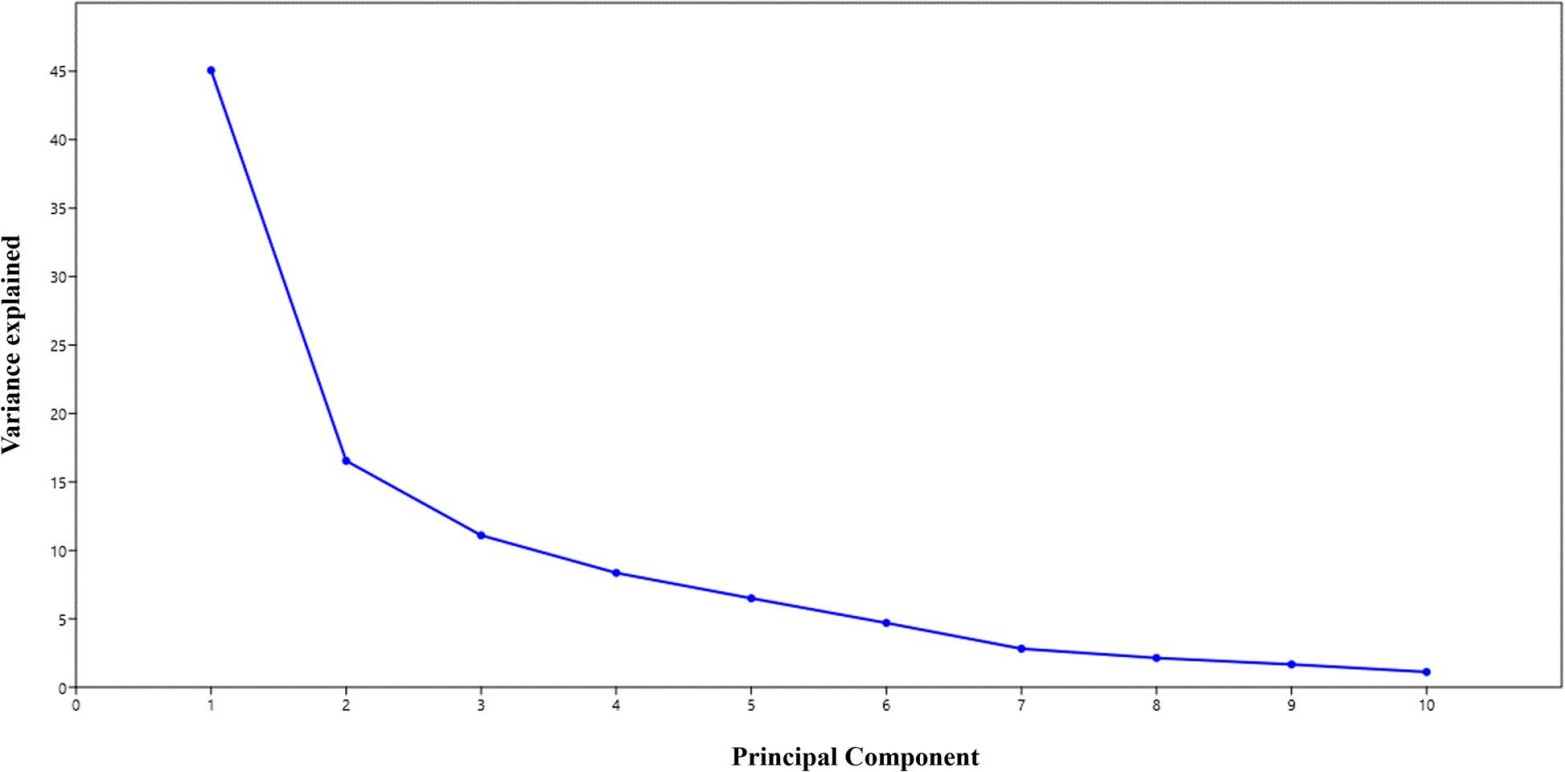
Scree Plot Showing Variance Explained by Principal Components. A scree plot illustrating the proportion of total variance accounted for by each principal component in the analysis of drought-related physiological and morphological traits among 118 indica rice doubled haploid lines and their parents. The first three components collectively explain over 72% of the observed variation, supporting their relevance for multivariate interpretation

**Fig. 6:**
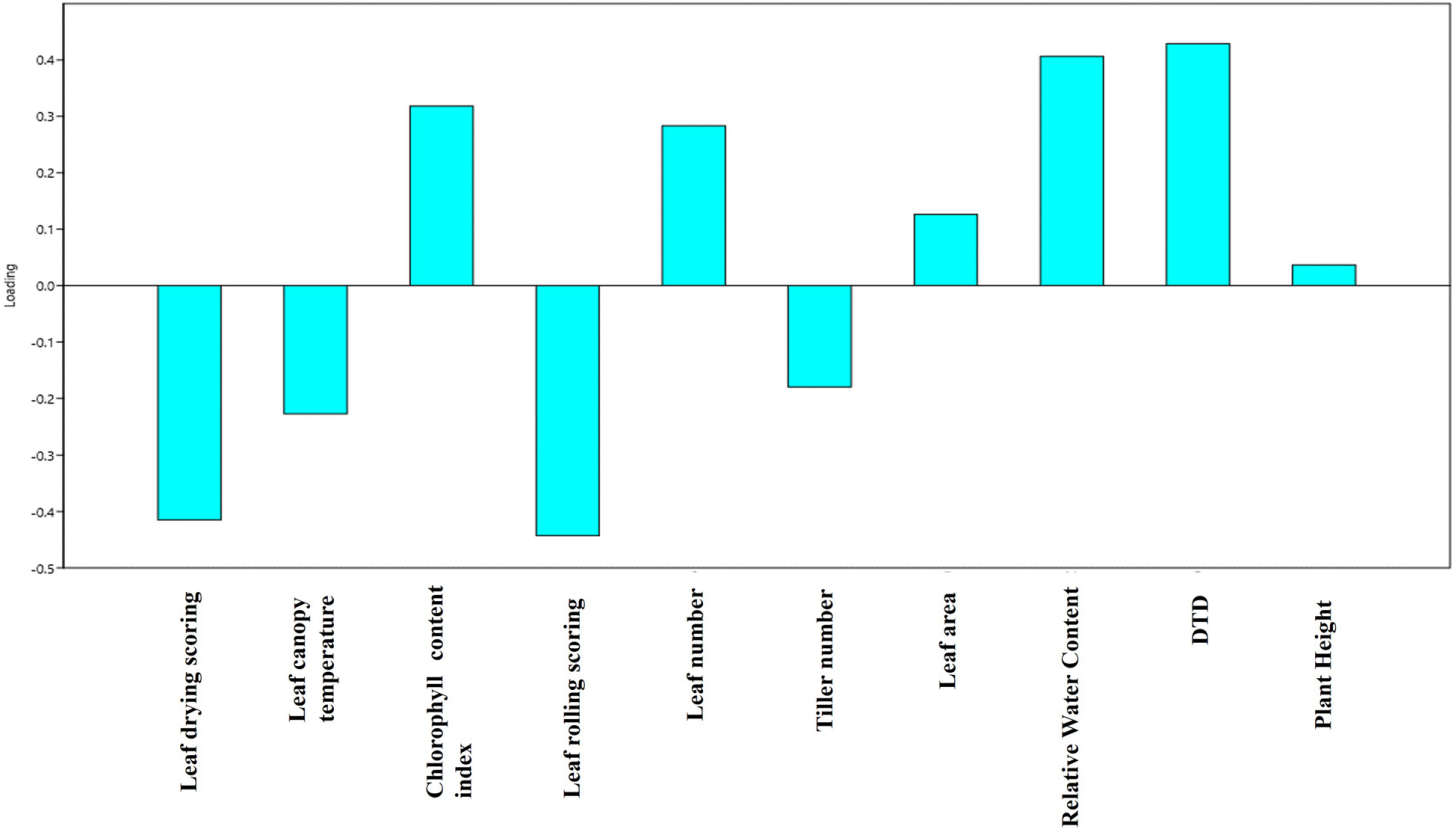
Loading Plot Depicting Contributions of Traits to Principal Component 1. A loading plot derived from principal component analysis (PCA), showing the relative contribution of each measured trait to the first principal component. Traits with the highest loading values, such as Drought Tolerance Degree (DTD), relative water content (RWC), and chlorophyll content index, are the most influential in explaining variance among the genotypes under study

**Fig. 7:**
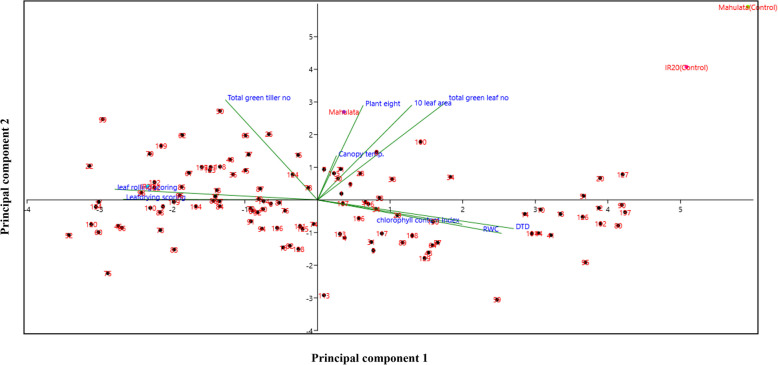
Biplot Representing Relationships Among Drought-Related Traits. A biplot from PCA displaying both genotypes and trait vectors, illustrating the degree and direction of association among variables such as DTD, RWC, chlorophyll content index, leaf rolling, and leaf drying scores. The proximity and angles between trait vectors reveal strong positive correlations among drought–tolerance–related traits, as well as negative associations between drought tolerance indices and stress scores

Principal Component Analysis (PCA) is a widely used multivariate statistical technique that reduces data dimensionality while capturing the major sources of variation among variables [21, 22]. In this study, PCA identified three principal components (PCs) with eigenvalues greater than or equal to 1, which together explained a substantial portion of the total variance. Specifically, PC1 accounted for 45.07% of the total variability, PC2 explained 16.54%, and PC3 contributed 11.09%, resulting in a cumulative variance of 72.71% explained by these three components (Table 3, Fig. 5). Among these, PC1 was the most influential, having the highest eigenvalue (Table 3). The variable loadings on PC1 (Fig. 6) revealed that the Drought Tolerance Degree (DTD) contributed most significantly, followed by relative water content (RWC) and chlorophyll content index. The PCA biplot (Fig. 7) further showed a strong positive correlation among DTD, RWC, and chlorophyll content, suggesting these traits collectively serve as effective indicators for drought tolerance screening.

**Table 3:** Principal component analysis of drought-related traits in indica rice: Eigenvalues of major principal components (the major principal component values are in bold)

PC	Eigenvalue	% Variance	Cumulative variance
1	**4.50715**	**45.071**	**45.071**
2	**1.65445**	**16.545**	**61.616**
3	**1.10941**	**11.094**	**72.71**
4	0.835506	8.3551	81.0651
5	0.649676	6.4968	87.5619
6	0.469732	4.6973	92.2592
7	0.281541	2.8154	95.0746
8	0.213713	2.1371	97.2117
9	0.166848	1.6685	98.8802
10	0.111972	1.1197	99.9999

Conversely, the leaf rolling and leaf drying scores formed angles with DTD, indicating negative correlations, consistent with their interpretation as symptoms increasing with drought severity. Because symptoms such as leaf rolling and leaf drying increase in genotypes that are less tolerant to drought stress, while DTD is higher in lines that maintain a better physiological status under drought. The principle underlying this association is largely physiological. Leaf rolling and drying are visible responses to water deficit caused by reduced turgor and cell dehydration. Genotypes with enhanced drought tolerance can maintain cellular water status, delay the onset of these symptoms, and thus display lower scores for leaf rolling and drying. These contrasting trait groupings clearly differentiated the drought-tolerant parent, Mahulata, from the susceptible parent, IR-20. Overall, the results support the use of DTD as a reliable indirect measure for selecting drought-tolerant rice lines.

### Hierarchical clustering analysis

**Fig. 8:**
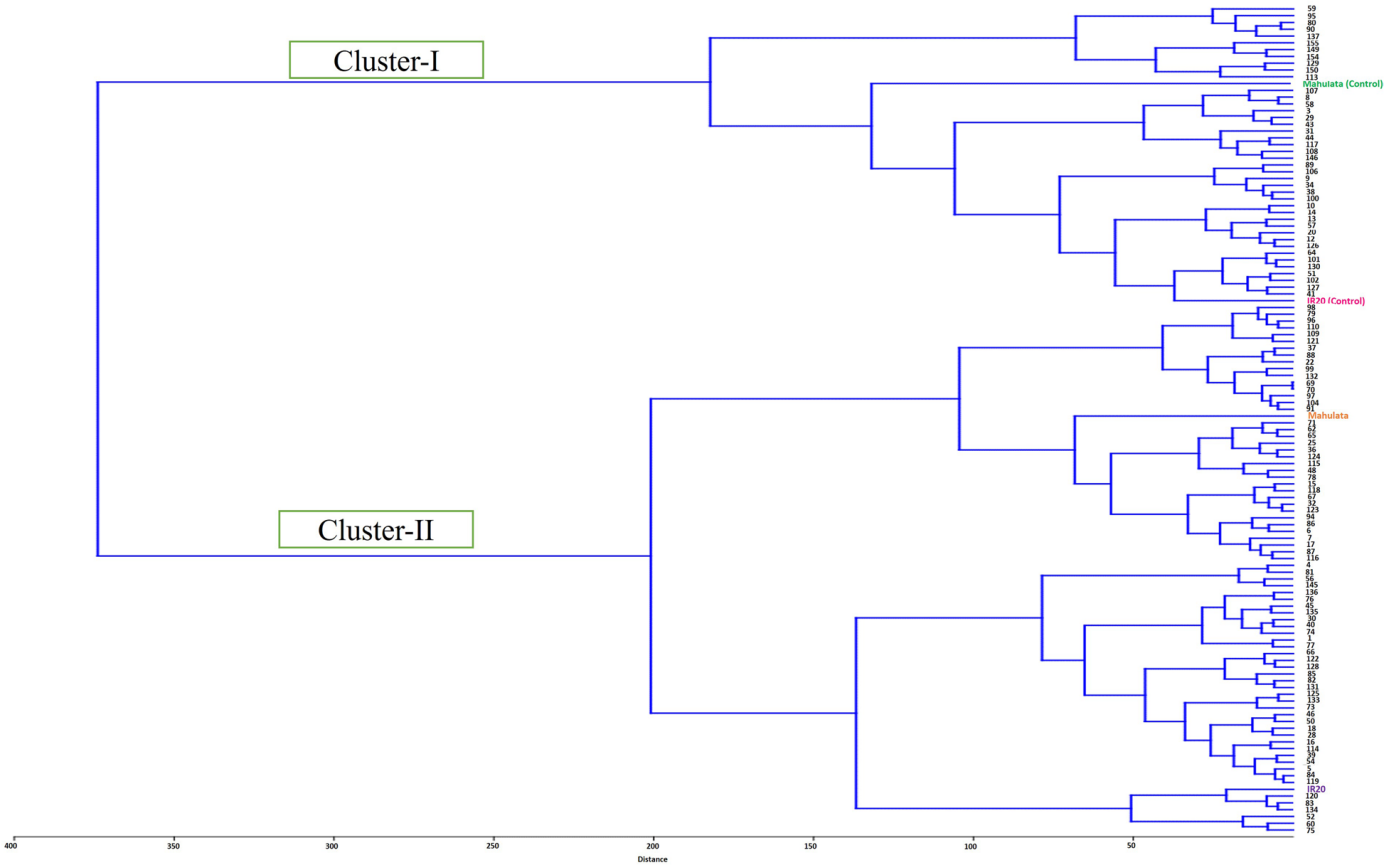
Hierarchical Clustering Dendrogram of Doubled Haploid Lines and Parents. A hierarchical clustering dendrogram grouping the 118 doubled haploid lines and two parental controls based on their physiological trait profiles under drought and control conditions. The analysis divides the population into two major clusters, separating drought-tolerant and susceptible genotypes, which is consistent with the PCA findings

To further validate the results from principal component analysis (PCA), hierarchical clustering analysis was performed on the 118 doubled haploid lines, along with the two parent varieties, under both drought-stress and well-watered control conditions. This analysis grouped the entire population into two major clusters based on a dissimilarity coefficient of 380 (Fig. 8). Cluster I comprised 43 lines, including both parent varieties grown under control (well-watered) conditions. Notably, this cluster contained the majority of drought-tolerant lines. Cluster II comprised the remaining 79 lines, along with both parents, which were subjected to drought treatment. The clear separation of clusters aligns with the observed physiological responses and supports the distinction between tolerant and susceptible genotypes under drought stress.

## Discussion

### Correlation of DTD values with physiological traits

Effective drought tolerance screening requires reliable, physiologically relevant indicators. Previous studies have shown that traits such as leaf tip drying and leaf rolling scoring are negatively associated with drought tolerance, where higher scores indicate greater stress sensitivity [23, 24]. Relative water content (RWC) is a direct physiological indicator of a plant’s water status and positively correlates with drought tolerance [25–27]. In this study, a strong positive correlation was observed between the Drought Tolerance Degree (DTD) values and RWC, and a negative correlation was found with leaf rolling and leaf tip drying scores. This confirms that DTD reflects plant water retention capacity and the extent of visible drought symptoms. Chlorophyll content index and leaf canopy temperature are also important proxies of drought stress responses. While lower canopy temperature and higher chlorophyll content observed in drought-tolerant lines correlate with better water status and transpiration cooling [28], the maintenance of such physiological advantages at severe soil moisture levels (~ 5–6%) should be interpreted with caution. In the extreme drought conditions, stomatal closure reduces transpiration, limiting evaporative cooling [24]. Thus, DTD likely reflects cumulative plant resilience integrating morphological and physiological traits rather than isolated mechanisms. The results demonstrated a positive association between DTD and chlorophyll content, as well as a negative correlation with leaf canopy temperature, reinforcing DTD as an indicator of physiological resilience under drought conditions. Collectively, these findings support the use of DTD as an integrative phenotype that correlates well with key physiological parameters such as RWC, leaf tip drying and rolling, chlorophyll content index, and leaf canopy temperature linked to drought tolerance, making it a practical tool for rapid screening.

### Role of the DTD method in drought-tolerance breeding

Morpho-physiological traits have long been utilized both as contributors to and indicators of drought tolerance in rice [25, 29]. This study advances this knowledge by validating the DTD method as a quantitative, rapid phenotypic screening tool in lowland rice seedlings. The strong concordance between DTD values and physiological traits such as RWC, chlorophyll content, and canopy temperature under drought stress implies that this method can reliably estimate drought tolerance levels in early growth stages.

Such rapid phenotyping is crucial for accelerating breeding programs, as early-stage selection prevents the need for resource-intensive evaluations later in the season. By providing a simple and reproducible metric, the DTD method can serve as an effective first-tier screen to identify promising genotypes for further evaluation under field conditions.

### Advantages and limitations compared with other screening approaches

The DTD method provides a rapid and quantitative alternative to traditional drought tolerance screening tools that often rely on subjective visual scoring (leaf rolling/drying) or labour-intensive biochemical assays (proline, ABA quantification) [30–39]. Unlike survival rate or grain yield-based assessments, which require long growth cycles and complex environmental controls, DTD enables early-stage phenotyping critical for accelerating breeding pipelines. Prior research has highlighted limitations of methods such as PEG-induced germination tests and proline content measurements due to environmental sensitivity and technical complexity [38, 40]. The DTD index’s reliance on green leaf retention offers a practical and scalable phenotyping strategy suitable for resource-constrained breeding programs [12, 30]. Multiple drought tolerance evaluation methods exist, including leaf rolling, survival rate, seed setting, grain yield, RWC, abscisic acid (ABA), and proline content analyses [30–39]. While these approaches are informative in nature, they face drawbacks such as being time-consuming, requiring complex biochemical assays, or being heavily influenced by variable environmental factors [40–44]. For example, PEG-mediated germination tests and proline quantification demand chemical reagents and specialized skills, limiting their scalability in breeding contexts. Leaf rolling scoring, while easy, is cultivar-dependent and subject to physiological variability unrelated to drought tolerance. Traits such as survival rate and grain yield are influenced by multiple stresses beyond drought, complicating their interpretation.

The DTD method overcomes many of these limitations. By focusing on measurements of green leaf retention at the seedling stage after a severe drought, it avoids the confounding effects of late developmental processes, disease pressures, and environmental fluctuations. This reduces noise and enhances the accuracy of drought tolerance estimation. Importantly, the method requires minimal technical expertise and no specialized equipment, making it cost-effective (Supplementary Table 1) and accessible to breeding programs with limited resources. Additionally, by quantifying a ratio rather than relying on qualitative scoring, the DTD approach reduces deviations caused by inherent differences in leaf morphology across genotypes, facilitating direct quantitative comparison. Its simplicity and efficiency make it conducive for high-throughput screening, which is essential for accelerating drought tolerance breeding.

The key limitation identified for the DTD method is its inapplicability at the harvest or maturity stage, due to natural leaf senescence and yellowing confounding the assessment of drought-induced damage. Therefore, the method is best suited for early-stage screening and should be complemented with field-based performance evaluations at later growth stages.

## Conclusions

In this study, the Drought Tolerance Degree (DTD) method, an effective tool for screening drought tolerance in indica rice, was validated. The analysis of 118 doubled haploid lines and their parents demonstrated strong positive correlations between DTD values and key physiological traits associated with drought resilience. Compared to other screening approaches, the DTD method proved to be cost-effective and practical, particularly for evaluating drought tolerance at the vegetative stage under controlled, severe drought stress conditions. Notably, this validation expands upon previous work conducted in japonica rice by applying the method to indica varieties and incorporating a comprehensive set of physiological parameters, including the relative water content (RWC), chlorophyll content index, tiller number, leaf tip drying and rolling scores, leaf area, canopy temperature, leaf number, and plant height. The use of a large doubled haploid mapping population and pot culture further strengthens the method’s applicability.

Overall, the DTD method provides a reliable, efficient, and scalable approach for screening drought tolerance in indica rice, making it a valuable tool for accelerating breeding programs aimed at developing drought-resilient cultivars. The DTD method likely reflects an integrative response encompassing morphological and physiological traits relevant to drought resilience. The integration of DTD phenotyping with molecular breeding tools that target key drought-responsive genes and regulatory pathways, such as NAC and DREB transcription factors, and ABA-dependent signaling, enhances the reliability and efficiency of drought tolerance screening. Integration of DTD-based screening with molecular markers and genomic selection strategies could further accelerate the development of drought-resilient rice cultivars adapted to increasingly variable climates.

## Supplementary Information

Supplementary Material 1

## Data Availability

The datasets generated and/or analyzed during the current study are not publicly available but are available from the corresponding author upon reasonable request.

## Abbreviations

DTD: Drought tolerance degree
DH: Doubled haploid
RWC: Relative water content
PCA: Principal component analysis
PC: Principal component
PEG: Polyethylene glycol
ABA: Abscisic acid
MM: Moisture meter
SES: Standard evaluation system
IR-thermometer: Infra-red thermometer
