# Supplementary material for: Assessment of Drought Tolerance Degree (DTD) method as a reliable tool for early-stage screening for drought tolerance in indica rice
Source: BMC Plant Biol. 2025 Nov 24;25:1630. doi: 10.1186/s12870-025-07591-7 (PMC12642344; doi:10.1186/s12870-025-07591-7)
Supplement: Supplementary file 1 — Supplementary Material 1 [file 12870_2025_7591_MOESM1_ESM.docx]

**Supplementary Table 1:** Comparative cost breakdown of different drought tolerance screening approaches in rice

| **Method** | **Direct Material Cost (per sample)** | **Labor/Skill Required** | **Specialized Equipment** | **Reference** |
| --- | --- | --- | --- | --- |
| **DTD method** | $0.05–$0.10 | Minimal | Ruler, spreadsheet |  |
| **Proline assay** | $2.50–$4.00 | Moderate | Spectrophotometer, reagents | https://www.elabscience.com/p/proline-pro-colorimetric-assay-kit--e-bc-k177-s |
| **ABA ELISA assay** | ~$6.00–$8.00 | Moderate/High | ELISA reader, kit | https://www.cusabio.com/ELISA-Kit/Plant-hormone-abscisic-acidABA-ELISA-Kit-62434.html |
| **PCR/molecular marker** | $5.00–$10.00 | High | PCR, gel docs, primers |  |
| **Leaf rolling/SES scoring*** | $0.02–$0.05 | Minimal | None |  |
| **PEG-based osmotic stress** | $0.25–$0.50 | Moderate | PEG, glassware, balances | https://www.himedialabs.com/in/tc740-polyethylene-glycol-peg-6000.html |
| **Field survival/yield^#^** | $0.05–$0.10 | Minimal/Season-long | Field, basic tools |  |

**Subject to physiological variability unrelated to drought tolerance*

*# Influenced by multiple stresses beyond drought*
